# Carbon Nanotube-Based Biosensors Using Fusion Technologies with Biologicals & Chemicals for Food Assessment

**DOI:** 10.3390/bios13020183

**Published:** 2023-01-24

**Authors:** Jinyoung Lee

**Affiliations:** Department of Green Chemical Engineering, Sangmyung University, Cheonan 31066, Republic of Korea; dorgly@smu.ac.kr

**Keywords:** carbon nanotube, electrochemistry, antibody, aptamer, microorganism, food pathogen

## Abstract

High-sensitivity sensors applied in various diagnostic systems are considered to be a promising technology in the era of the fourth industrial revolution. Biosensors that can quickly detect the presence and concentration of specific biomaterials are receiving research attention owing to the breakthroughs in detection technology. In particular, the latest technologies involving the miniaturization of biosensors using nanomaterials, such as nanowires, carbon nanotubes, and nanometals, have been widely studied. Nano-sized biosensors applied in food assessment and in in vivo measurements have the advantages of rapid diagnosis, high sensitivity and selectivity. Nanomaterial-based biosensors are inexpensive and can be applied to various fields. In the present society, where people are paying attention to health and wellness, high-technology food assessment is becoming essential as the consumer demand for healthy food increases. Thus, biosensor technology is required in the food and medical fields. Carbon nanotubes (CNTs) are widely studied for use in electrochemical biosensors. The sensitive electrical characteristics of CNTs allow them to act as electron transfer mediators in electrochemical biosensors. CNT-based biosensors require novel technologies for immobilizing CNTs on electrodes, such as silicon wafers, to use as biosensor templates. CNT-based electrochemical biosensors that serve as field-effect transistors (FET) increase sensitivity. In this review, we critically discuss the recent advances in CNT-based electrochemical biosensors applied with various receptors (antibodies, DNA fragments, and other nanomaterials) for food evaluation, including pathogens, food allergens, and other food-based substances.

## 1. Introduction

**Figure 1:**
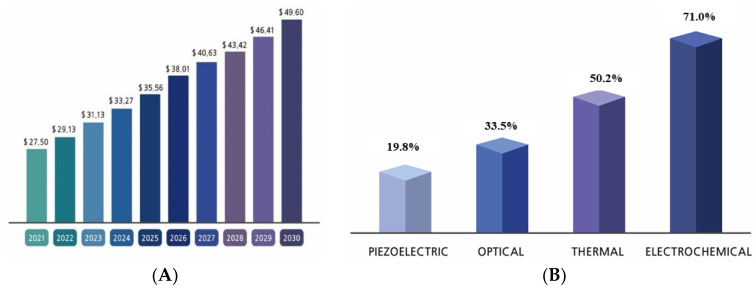
The world market in biosensors. (**A**) Biosensor market size; (**B**) Types of biosensors in industrial market share [1].

The global biosensor market is rapidly growing owing to emerging diseases, and the adoption of innovative technologies, such as artificial intelligence and the internet of things, is common in the era of the fourth industrial revolution [1]. Biosensors are widely used to detect and monitor biomolecules in various industries, and this usage has grown rapidly, particularly in the era of COVID19, as biosensors are mainly used to detect coronavirus in patients. Chip-based biosensors are mainly utilized to detect virus spread among people. As shown in Figure 1A, the market for biosensors as of 2021 was 27.50 billion USD and is expected to expand to 49.60 billion USD by 2030. Biosensors can be categorized as piezoelectric, optical, thermal, and electrochemical depending on the sensor signal utilized; among these, 71% are electrochemical biosensors (Figure 1B).

Monitoring food safety and maintaining food stability are essential in the food industry. However, food regulators and manufacturers are making various efforts to reduce food risk, such as implementing good manufacturing practices, controlling hazards, and ensuring critical control points [2]. Recently, food allergy cases have been increasing year by year and have put a significant burden on human health and life over the past few decades [3]. People with hypersensitivity syndrome, which causes hyperimmune reactions, can have adverse health effects and even die if the consumed food contains harmful microorganisms, pathogens, and chemicals [4]. Consequently, accidental consumption of foods containing these toxic substances and the frequency of such consumption cannot be ignored [5], as this can harm the food industry. Based on many statistical studies, food allergies have become a common chronic disease in children and affect approximately 8% of children, and even up to 10% in developed countries [6,7]. Food allergens generally cause immune sensitivity to specific proteins or glycoproteins (antigens), such as peanut proteins [8]. The hypersensitivity reactions induced by these food allergens harm our bodies, causing diarrhea, nausea, dermatitis, and hives. In severe cases, such reactions can be dangerous to life [9]. Therefore, improving food-safety monitoring technologies is conducive to securing national competitiveness and public health.

These days, there is a growing demand for biosensor technology that can secure the safety of processed foods, monitor them in real time, and reliably detect food pollutants in the food industry [10]. A biosensor detects biological materials, such as metabolites (glucose, lactic acid, protein, etc.), DNA, and microorganisms, and converts biological signals into electricity, coloration, waves, and other detectable signals. These biosensor technologies are expected to evolve further in the era of the fourth industrial revolution as the reaction time is shortened and the selectivity and sensitivity of the biosensors is enhanced. A biosensor consists of a template forming the sensor shape, sensor receptor combining biomaterials, and signal converter reading the binding of biomaterials into an easy-to-understand signal. Bio receptors are enzymes, antibodies, antigens, cells, and DNA. A biosensor is an integrated receptor–transducer device, converting a biological response into an electrical signal [11], and can be applied to various fields, such as medicine, the environment, and food, which are directly related to human health; they are simpler, require shorter detection times, and exhibit higher selectivity and sensitivity than other analytical devices. Currently, biosensors are widely used in academia, industry, and laboratories [12,13,14]. They quantitatively analyze target biomaterials by changing the strength of sensor signals depending on the concentration of detected substances and the presence of targets in a specific space. Biosensors can detect different biomaterials associated with the medical field (blood, urine, saliva, tears, and sweat), the environment (food hygiene bacteria, fungi, and food allergy-causing proteins), and farming (fertilizers, nutrients, and plant physiological samples) [15,16]. In addition, biosensors are effective in high-throughput screening tests, food and medicine safety, agriculture, environmental protection, and pharmacology [17].

Furthermore, developing the biosensor technology is crucial for improving microfluidics [18]. A microfluidic channel safely transfers samples to receptors in a biosensor and enables the miniaturization of the biosensor. The microchannel technology for biosensors is well-developed because it prevents the contamination of receptors [19,20] and allows for the creation of various biosensor designs for different applications.

Electrochemical biosensors convert biological signals into electrochemical signals. These biosensors are widely studied and utilized in multiple applications owing to their excellent selectivity, high sensitivity, and simultaneous detection capability in the reaction between target materials and biosensor receptors [21,22,23,24]. Electrochemical biosensors consist of working, reference, and counter electrodes, and the sensor receptors are the working electrodes. The types of electrochemical biosensors involve potentiometric, amperometric, impedimetric, conductometric, and voltammetric transduction mechanisms depending on the electrochemical signals they receive [21]. A potentiometric biosensor detects a target material based on the amount of charge accumulated in the working electrode compared with a reference electrode in response to a reaction between a target material and a sensor receptor at an initial zero current value. The working electrode can detect the current generated through the electrochemical oxidation or reduction in the electroactive species. Moreover, the working electrode obtains a constant voltage with respect to the reference electrode [25,26]. A conductometric biosensor detects a change in conductivity between two electrodes due to a reaction between a target material and a sensor receptor. Both conductometric and impedimetric biosensors are frequently utilized to analyze metabolites in living biological models [27]. The impedimetric biosensor uses the electrical impedance generated by the electrode/electrolyte contact in a reaction between a target material and a sensor receptor. It uses a function graph of the frequency generated when a low-amplitude AC voltage, which is applied to the sensor electrode [28]. A voltammetric biosensor detects the analyte using the voltage change caused by a reaction between a target material and a sensor receptor [29].

Carbon is a potential electrical material because it is inexpensive and has bio-friendly properties. The studies on carbon nanotube (CNT)-based biosensors have actively devolved since the discovery of CNTs [30,31]. CNTs are applied as electrodes in biosensors or used as electron transfer mediators [32]. CNTs are cylinders in which a single or multiple graphene sheets are rolled up, and carbon atoms form a hexagonal mesh. Each carbon atom in a CNT has a single lone pair of electrons, allowing CNTs to transfer electrons derived from an electrical signal as a response by the sensor system. CNTs include single-walled carbon nanotubes (SWCNTs) and multi-walled carbon nanotubes (MWCNTs). SWCNTs have a higher thermal conductivity, smaller diameter, and higher current transfer capacity than MWCNTs [33,34,35]. Both SWCNTs and MWCNTs are applied to various sensors owing to their similar electrochemical characteristics. SWCNTs are applied to small semiconductors owing to their smaller diameter than MWCNTs. Compared with a metal SWCNT, a high-sensitivity semiconducting SWCNT has many electrochemical advantages because all the carbon atoms are located on the surface and have high mobility; hence, it is suitable for sensor devices [36,37]. Compared to SWCNTs, MWCNTs are robust and short in length while they have a relatively high concentration gradient. In particular, when MWCNTs are used in a biosensor, it is advantageous to have a high surface area so that more biosensor receptors can be attached to the MWCNTs when immobilized on the electrode surface. In the manufacturing process of CNT-based biosensors, two electrodes in an electrochemical biosensor should be connected by CNTs immobilized onto the sensor device. The CNTs are assembled [38] by mainly employing the drop-casting or dielectrophoresis (DEP) method that supplies voltage to the anode.

In this study, we investigate and discuss the concepts of novel CNT-based electrochemical biosensors applied with various receptors (antibodies, DNA fragments, nano-metal particles, and other chemical nanomaterials) for food safety assessment, including pathogens, food allergens, vital metabolites, and nutrients, in the food industry in the era of the fourth industrial revolution.

## 2. Concepts of CNT-Based Electrochemical Biosensor

A CNT-based electrochemical biosensor has the advantage of label-free high-sensitivity detection owing to its high electrical conductivity [39]. Studies on CNT-based electrochemical biosensors have been widely conducted in the fields of the environment [40,41], agriculture [42,43], food [44], energy [45,46], and medicine [47]. Mainly, it is advantageous to detect harmful microorganisms or food allergens using the sensitivity of CNTs in the food industry. In the medical field, portable biosensors are used for blood sugar and pulse measurements and wearable biosensors to detect NaCl and lactic acid in sweat [48,49]. CNT-based biosensors are divided into four categories depending on the signal being converted: electrochemical, optical, semiconductor, and calorimetry biosensors [50]. Among them, the electrochemical biosensor is widely used. It has excellent sensor capacity and sensitivity, is of a small size, has low manufacturing costs, rapid response time, and a wide detection range (DR) [51]. CNT-modified biosensors require a receptor assembly process such as an enzyme, DNA fragment, living cell, antibody, and other chemical materials. There are two CNT modification processes: a covalent process sharing electrons through a chemical reaction, and a non-covalent process such as adsorption on the CNT surface through a physical method.

Functionalization of CNT for the biosensor using physical and chemical methods depends on the reactant temperature and pH. Specifically, CNT functionalization using a protein enzyme as a biosensor acceptor has a great influence on the reaction of the three-dimensional structure of the enzyme with the target substance. In this case, the structure of the enzyme immobilized on CNT reacts very sensitively to temperature, ionic strength, pH, and substrate concentration. In addition, though the biosensor receptor is an antibody, not enzyme, the optimal temperature and pH are required in sensor reaction due to the protein structure of antibody.

### 2.1. Covalent Bonding Modification of CNT for Receptor Immobilization

**Figure 2:**
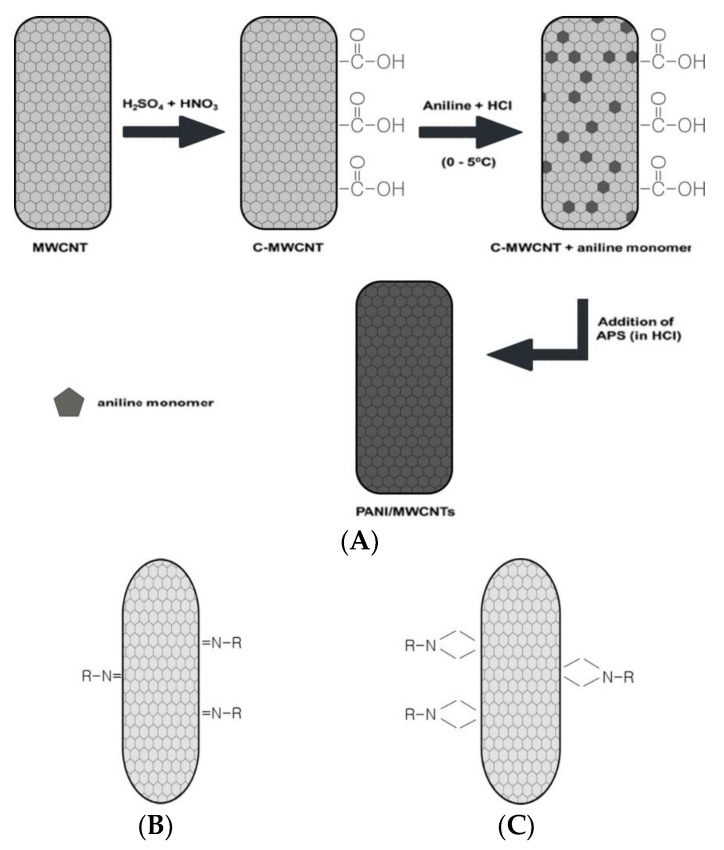
The scheme of functionalized CNTs. (**A**) The chemical process for forming covalent bond on the CNTs through oxidation process [53]; (**B**) The chemical process for forming covalent bond on the CNTs through a photochemical oxidation process; (**C**) The chemical process for forming covalent bond on the CNTs through an electrochemical oxidation process [57].

There are two main covalent chemical processes for CNT functionalization in CNT-based biosensors. One method is utilizing the carboxyl group (COOH) present at a CNT terminal or an additional reaction to produce a functional group that enables covalent bond formation on the CNT wall. The CNT oxidization reaction is the most common method for functionalizing the CNT surface with COOH [52]. The CNT surface is oxidized with HNO_3_ and H_2_SO_4_ in a 1:3 ratio for approximately 6 h to form a chemical functional group (Figure 2A) [53]. The COOH group generates an amide bond with an ester so that a covalent bond can immobilize the bioreceptor on the CNT.

Atoms with non-covalent electron pairs are bonded to CNTs to increase electronegativity, which increases hydrogen bonding and eventually increases the dispersibility and solubility of CNTs [54]. The COOH group on the surface of the modified CNTs can create a chemical environment to efficiently immobilize the bioreceptors using their amine groups (NH_2_). Here, N-ethyl-N’-(3-dimethylaminopropyl) carbodiimide (EDC) is one of the most common reagents for immobilization using covalent bonding, and it reacts with COOH groups to produce intermediate 0-acylisourea esters that can easily bind to a primary NH_2_ [55]. However, the natural electrochemical function of CNT is likely to be weakened since the CNT surface is chemically altered. Therefore, there is a concern that applying CNTs to biosensors may dilute the existing electrochemical properties to some extent [56]. In addition, when oxidizing a CNT surface, the entire CNT reacts with an oxidizing solvent, and some by-products are generated during the reaction. Furthermore, the overall structure of CNT could be changed because the volume and length of the CNT may be reduced during the oxidation of CNTs [57]. Such changes in covalent bonds due to oxidized CNTs are demonstrated by studying photoluminescence and Raman scattering [58].

The other methods of CNT covalent bonding are photochemical and electrochemical functionalization. The photochemical functionalization with photoirradiation produces reactive species such as nitrenes in the reaction on the CNT wall (Figure 2B) [57]. A CNT electrode contained in a solution with an appropriate reactant generates a constant potential or current during the electrochemical process (Figure 2C) [57]. The capacity of biosensors manufactured with covalently bonded CNTs has been studied to detect and analyze target materials. An electrochemical biosensor composed of horseradish peroxidase (HRP)-encapsulated protein nanoparticles (HEPNP) was fabricated for the sensitive and selective detection of H_2_O_2_ [59]. The detection signal was current, and the coincidence rate was 97.6% compared to 33.6(4.3) % (w/w) from the standard comparison detection method [60]. Another study modified an electrochemical biosensor using MWCNT and γ-aminobutyric acid (GABA) to detect HRP [61]. Cyclic voltammetry (CV cycle) showed redox peaks during the Fe(III)/Fe(II) redox reaction at pH 7.0. The measured heterogeneous electron transfer rates, surface coverage, and Michaelis–Menten constants of fixed HRP were 1.11 s^−1^, 3.029 × 10^−9^ mol/cm^2^, and 0.23 mM, respectively. The biosensor showed good efficiency for finding H_2_O_2_ with a broad linear range from 2.0 × 10^−7^ M to 2.81 × 10^−4^ M and a detection limit of 0.13 μM.

### 2.2. Non-Covalent Bonding Modification of CNT for Receptor Immobilization

**Figure 3:**
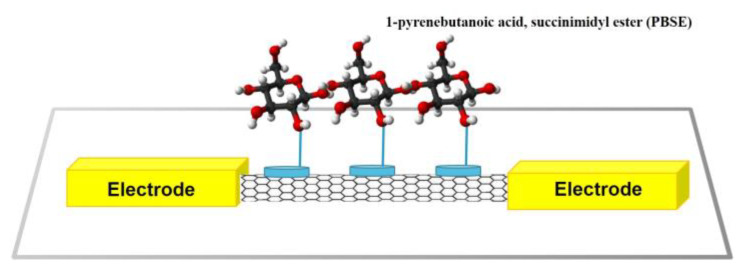
Noncovalent bond-based functionalization of SWCNTs using 1-pyrenebutanoic acid, succinimidyl ester (PBSE) [64,65].

The CNT adsorption using non-covalent bonds is performed through interactions such as p-p, CH-p, and van der Waals between the medium and CNT. The hydrophobic portion of the medium adsorbed to CNT is held by interacting with the CNT wall. The hydrophilic portion solubilizes in the solvent and subsequently induces binding to the sensor receptor [52]. CNT functionalization using non-covalent bonds has the considerable advantage of attaching the desired chemical functional group to the CNT surface while maintaining the constant p-system characteristics of the carbon net layer inside the CNT compared to CNT functionalization using covalent bonds [57]. The functionalization of CNTs using non-covalent bonds is achieved by the π–π interactions, which can be obtained through electrostatic interaction between aromatic molecules and CNTs. CNTs with high hydrophobicity are not well-dispersed in a general hydrophilic solvent and, thus, constitute a significant obstacle in the application of biosensors. Therefore, the solubility of CNT is improved by using a hydrophobic solvent such as an aromatic polymer or a surfactant, leading to the highly efficient adsorption of the medium to the CNT surface [55]. In the non-covalent CNT functionalization, derivatives of amine-reactive pyrene [62] and pyrene-conjugated glycodendrimers [63] were used for the CNT functionalization. Figure 3 shows the formation of a chemical functional group on the surface of SWCNT by immobilizing a 1-pyrenebutanoic acid, succinimidyl ester (PBSE) medium forming a non-covalent bond with SWCNT, and the immobilization of proteins with the modified SWCNT wall [64,65].

**Figure 4:**
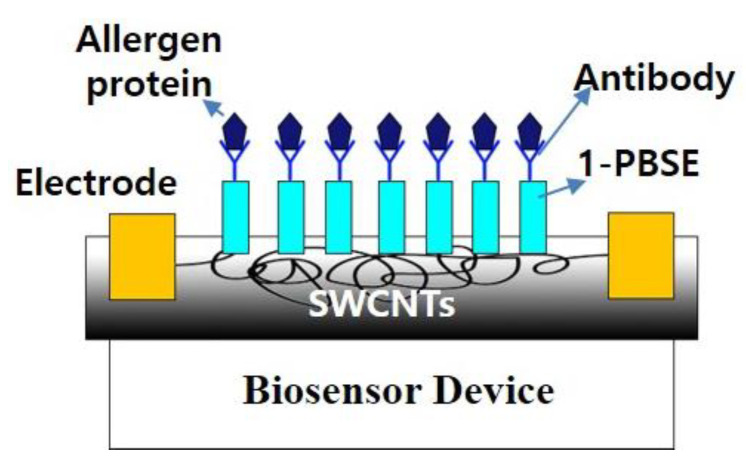
Scheme of SWCNT-based nano-biosensor for the detection of allergen-derived proteins in processed foods [67].

A biosensor with non-covalent CNT functionalization has been studied for various purposes. The non-covalent functionalization was used to manufacture an electrochemical sensor based on a MWCNT-polyazocarmine B nanofilm to detect nitrogen monoxide (NO). The resulting sensor exhibited high sensitivity, stability, reproducibility, and selectivity, indicating that it effectively monitored and detected NO [66]. As another example, an antibody-based biosensor was developed using non-covalently modified SWCNT to detect *Yersinia enterocolitica*, a harmful microorganism that may exist in the food named “Kimchi”. SWCNT-based biosensors were fabricated by immobilizing anti-*Yersinia* antibodies (pAbs) on PBSE. The sensor generated an electrochemical signal using ∆R and was calculated by performing linear sweep voltammetry (LSV). The specific binding reaction between the antibody (pAbs) and *Y. enterocolitica* was confirmed by indirect enzyme-linked immunosorbent assay (ELISA), while the target bacteria separated from the white Kimchi products was identified through the 16SrDNA sequence. The ∆R for detecting *Y. enterocolitica* has significantly increased in an antibody-based SWCNT biosensor. The optimal concentration range of enterocolitica detection was 10^4^–10^6^ CFU/mL, whereas the limit of detection (LOD) of *Y. enterocolitica* in Kimchi products was 10^4^ CFU/mL [67]. In addition, the antibody-based biosensor was developed using non-covalent modified SWCNT to detect Arachis hypogea 1 (Ara h1), a peanut allergy-causing protein (Figure 4).

The sensor signal was calculated by performing LSV measurements using ∆R and a potentiostat. The DR could be measured in a wide range of Ara h1 concentrations, 1.0–10^5^ ng/L. The biosensor was exposed to various foods containing food additives and could successfully distinguish the peanut components [68]. An Apt (a specific DNA fragment with a high affinity to target materials)-based biosensor functionalized with SWCNT by the non-covalent method was developed to detect oxytetracycline (OTC), which is an antibiotic widely used in the agricultural and fisheries industries. A novel flexible biosensor device was manufactured by a high-rate nanoscale process using a directed assembly of SWCNTs. Since this environmental biosensor introduces a real-time continuous detection system, continuous OTC detection can be performed using resistance signal monitoring [69].

### 2.3. Sensor Acceptors in CNT-Based Electrochemical Biosensor

The modified CNT-based electrochemical biosensor determines the sensor receptor according to the target material. Representative sensor acceptors include redox enzymes, immune antibodies, and DNA fragments such as Apts. The modified CNT-based enzyme biosensor can detect in continuous real-time by reversibly reacting the target materials. The novel CNT biosensors have been steadily developed using various sensor receptors with electrochemical systems, as presented in Table 1 [70].

**Figure 5:**
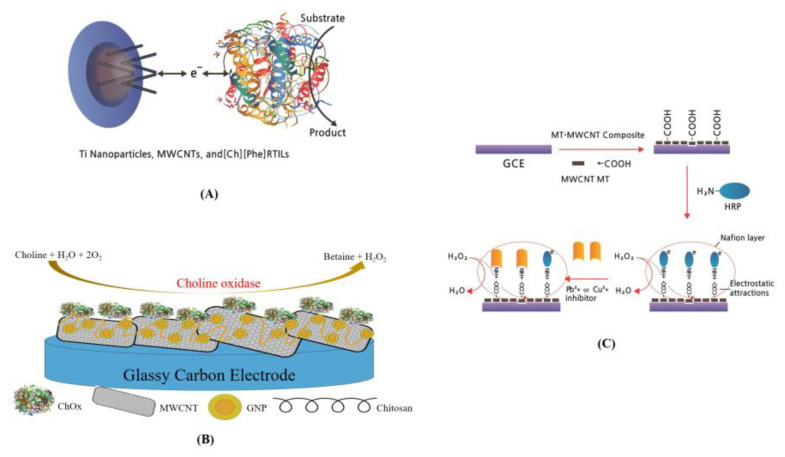
CNT-based biosensor with enzyme as a bioreceptor. (**A**) Scheme of SWCNT-based nano-biosensor using GC screen-printed electrode (SPE) was manufactured modified with MWCNTs, TiO_2_ nanoparticles, and newly produced ionic liquids (RTILs) for glucose detection [72]; (**B**) Scheme of amperometric biosensor using ChOx/(GNP)4/MWCNT/GCE for choline determination [81]; (**C**) Stepwise amperometric biosensor fabrication process and immobilized horseradish peroxidase inhibition in metal ion solution [82].

The use of enzymes as receptors for biosensors has been widely developed for decades with a wide range of applications. Enzyme-based biosensors are in the spotlight as an innovative detection technology because their accuracy is proven in qualitative and quantitative analysis. Owing to the advantages of enzyme-based biosensors, such as high sensitivity, specificity, portability, low operating cost, miniaturization, and point-of-care diagnostic tests, their applications are expanding in various industries, including medicine, food, and the environment [71]. Zappi et al. [72] developed an electrochemical biosensor using a new modified electrode based on glassy carbon (GC). In particular, the developed GC screen-printed electrode (SPE) was fabricated and modified with MWCNTs, TiO_2_ NPs, and newly produced ionic liquids (RTILs) that are eco-friendly at room temperature. The green RTIL immobilized the enzyme on the electrode surface and had its electrical conductivity, which made it an excellent electron transfer medium. The developed biosensor detected glucose with a LOD of 3 × 10^−4^ nM and a DR of (1.0–15) × 10^−3^ µM (Figure 5A). CNT-based electrochemical biosensors using glucose oxide for detecting glucose are the most widely used biosensors. These biosensor applications have been expanded from glucose detection [72,73] to the detection of fruits [74] and the decay of fish [75] in the food industry and medical detection kits. The sensor capacities (sensitivity, selectivity, stability, reproducibility, etc.) have been rapidly improved with the large adapted industrial area. Chen et al. [76] fabricated a highly flexible MWCNT-biosensor using carbonized silk fabric (MWCNTs/CSF) by modifying it with Pt microspheres for glucose detection. This biosensor’s advantage was electrode flexibility, facilitating its application in wearable electronic devices. The results showed a high sensitivity for hydrogen peroxide (H_2_O_2_) in glucose detection after Pt microsphere modification by electrodeposition.

**Table 1:** Summary of CNT-based electrochemical biosensors with various bio-acceptors (enzyme, antibody, and DNA fragment including aptamer).

Bio-Acceptors	Analyte	Method	RD	LOD	DR	Ref.
Enzyme	Glucose	AMP	GOx	3 × 10^−4^ nM	(1–15) × 10^−3^ µM	[72]
	Glucose	AMP	GOx	5 × 10^−5^ nM	(0–5) × 10^−3^ µM	[76]
	Glucose	AMP	GOx	2.99 × 10^−6^ nM	(3–14) × 10^−3^ µM	[77]
	Alcohol	AMP	ADH	3.3 × 10^−3^ nM	(12.5–100) × 10^−3^ µM	[72]
	Ethanol	AMP	ADH	1 × 10^−5^ nM	(1–5) × 10^−4^ µM	[78]
	L-malic acid	AMP	MDH	6 × 10^−5^ nM	(0–120) × 10^−6^ µM	[79]
	Xanthine	AMP	XOx	1.2 × 10^−7^ nM	(2–86) × 10^−6^ µM	[80]
	Choline	AMP	COx	6 × 10^−7^ nM	(3–120) × 10^−6^ µM	[81]
	Lead ions	AMP	HRP	2.5 µg/l	0.092–0.55 mg/l	[81]
	Copper ions	AMP	HRP	4.2 µg/l	0.068–2 mg/l	[82]
Antibody	TrT	AMP	Ab	33 pg/mL	0.1–10 ng/mL	[83]
	DVN	AMP	Ab	35,000 pg/mL	1000–2500 ng/mL	[84]
	ZEA	AMP	Ab	0.15 pg/mL	0.001–0.1 ng/mL	[85]
	CA19-9	SWV	Ab	0.163 pg/mL	0.001–100 ng/mL	[86]
	HER2	IS	Ab	7.400 pg/mL	10–110 ng/mL	[87]
	CA19-9	IS	Ab	0.35 U/ml	NR	[88]
DNA fragments including aptamer	ALF	DPV	DNA	3.5 fM	10^−14^–10^−8^ M	[89]
	HB-DNA	DPV	DNA	2.5 fM	10^−14^–10^−10^ M	[90]
	LNC-RNA	DPV	DNA	42.8 fM	10^−14^–10^−7^ M	[91]
	MicroRNA 21	DPV	DNA	0.01 fM	10^−17^–10^−6^ M	[92]
	SSE	DPV	DNA	17 × 10^−6^ fM	NR	[93]
	S-CML	IS	DNA	1 fM	10^−15^–10^−6^ nM	[94]
	Thrombin	DPV	DNA	0.08 pM	0.001–4 nM	[95]
	Silver ions	SWV	DNA	1500 pM	2–100 nM	[96]
	HER2	IS	DNA	50 fg/ml	0.1 pg/mL	[97]

Abbreviation: Ab: antibody; ADH: alcohol dehydrogenase; ALF: Anthrax lethal factor; AMP: Amperometry; CA19-9: pancreatic cancer biomarker protein; Cox: choline oxidase; DR: detection range; DVN: Dengue virus; GOx: glucose oxidase; HB-DNA: Hepatitis B virus genomic DNA; HER2: human epidermal growth factor receptor 2; HRP: horseradish peroxidase; IL: interleukin; IS: Impedance spectroscopy; LOD: limit of detection; MDH: malate dehydrogenase; LNC-RNA: Long non-coding RNA; NR: not reported; NS1 protein: Non-structural protein; RD: receptor details; Ref.: reference; S-CML: Sequence specific to chronic *myelogenous leukemia;* SSE: Sequence specific to *E. coli*; SWV: Square wave voltammetry; TrT: cardiac biomarker Troponin T; XOx: xanthine oxidase; ZEA: zearalenone.

The MWCNT-biosensor obtained a high glucose sensitivity of 288.86 µA/mM·cm^2^ with an excellent linear range from 0 to 5 mM. A nano-interfaced amperometric biosensor was developed for the rapid and accurate assessment of glucose in cancer cells by Madhurantakam et al. [77]. They demonstrated a hybrid nano-interface comprising a CNT and graphene (GR) blend that increase direct electron transfer by enhancing the surface area of the working electrode. Detection of the used glucose was obtained at predetermined time intervals in human pancreatic cancer cells, MiaPaCa-2. Wilson et al. demonstrated the advantage of polytyramine (PT) in a biosensor platform, which forms an easy electropolymerizable film for combining alcohol dehydrogenase (ADH) and a reduced nicotinamide adenine dinucleotide (NADH) as an electron transfer mediator at a low overpotential, which is essential to design high-performance ethanol biosensors [78]. PT has been adsorbed onto the surface of an MWCNT-modified GC electrode via DEP in which tyramine was oxidized in 0.1 M phosphoric acid (H_3_PO_4_) by cycling the potential over the range of −400−1300 mV with an Ag/AgCl reference electrode. This biosensor detected ethanol with a sensitivity of 4.28 ± 0.06 μA/mM·cm^2^, a linear range of 0.1−0.5 mM, and a LOD of 10 μM. Magar et al. fabricated an MWCNT-based electrochemical biosensor for choline determination [81], which was composed of MWCNTs, gold NPs (GNPs), and a natural biocompatible polymer called chitosan (Chit). Chit-MWCNTs were dropped on the surface of a GC electrode (GCE), followed by immobilizing GNP and choline oxidase (ChOx) by glutaraldehyde crosslinking. The developed ChOx/(GNP)_4_/MWCNTs/GCE-based biosensor showed a linear response to choline from 3 to 120 µM while the sensitivity and LOD were 204 µA/mM·cm^2^ mM and 0.6 μM, respectively (Figure 5B). MWCNTs conjugated with horseradish peroxidase (HRP)-based biosensor was developed by Moyo et al. [82] for trace metal ions detection. HRP was immobilized on maize tassel-MWCNTs (MT-MWCNTs) via electrostatic interactions. Trace metal ions were detected by the negative reaction of H_2_O_2_ with HRP immobilized in a standard solution. After the sensing conditions were optimized, the MWCNT-based biosensor showed DRs of 0.092–0.55 mg/L, and 0.068–2 mg/L for Pb^2+^ and Cu^2+^, respectively, LODs of 2.5 μg/L for Pb^2+^ and 4.2 μg/l for Cu^2+^ (Figure 5C).

**Figure 6:**
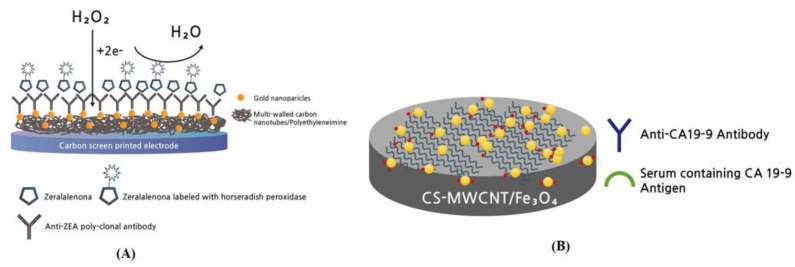
CNT-based biosensor with antibody as a bioreceptor. (**A**) Scheme of an electrochemical immunosensor to determine zearalenone (ZEA) mycotoxin [85]; (**B**) Scheme of bio-nanocomposite-based highly sensitive and label-free electrochemical immunosensor using Anti-AbsCA19-9/CS-MWCNT-Fe_3_O_4_ immune-electrode fabricated on glassy carbon electrode (GCE) for the detection of carbohydrate antigen 19–9 (CA19-9) [86].

As biosensor receptors, polyclonal, monoclonal, and recombinant antibodies have been developed for immune diagnosis and biomarker detection. Biosensors with high specificity and stability of antibodies are in the spotlight as self-testing biosensors for coronavirus detection and many other applications. Riberi et al. developed an electrochemical immunosensor to determine zearalenone (ZEA), a mycotoxin in corn crops [85]. These MWCNTs were based on a composite, prepared from anti-ZEA poly-clonal antibody bonded to gold NPs immobilized on MWCNTs/polyethyleneimine dispersions with carbon screen-printed electrodes (CSPE) in the electrochemical transduction stage. High sensor capacities were accomplished for the MWCNT biosensor with a linear DR of 10^−4^–10^−1^ ng/mL and a LOD and SC50 of 0.15 pg/mL and 2 pg/mL, respectively (Figure 6A).

Kalyani et al. manufactured an antibody-conjugated NP-based electrochemical label-free immuno-biosensor with high sensitivity for endometriosis diagnostics [86]. MWCNTs and magnetite NPs (MWCNTs–Fe_3_O_4_) with chitosan were applied to the fabrication of a bio-nanocomposite to immobilize the monoclonal specific antibody using a cross-linking method with glutaraldehyde for carbohydrate antigen 19–9 (CA19–9) detection. This conjugated antibody-based biosensor with a well-characterized Anti–AbsCA19–9/CS–MWCNTs–Fe_3_O_4_ immune-electrode fabricated on GCE had a high sensitivity of 2.55 µA/pg·cm, a LOD of 0.163 pg/mL, and DR from 1.0 pg/mL to 100 ng/mL (Figure 6B).

**Figure 7:**
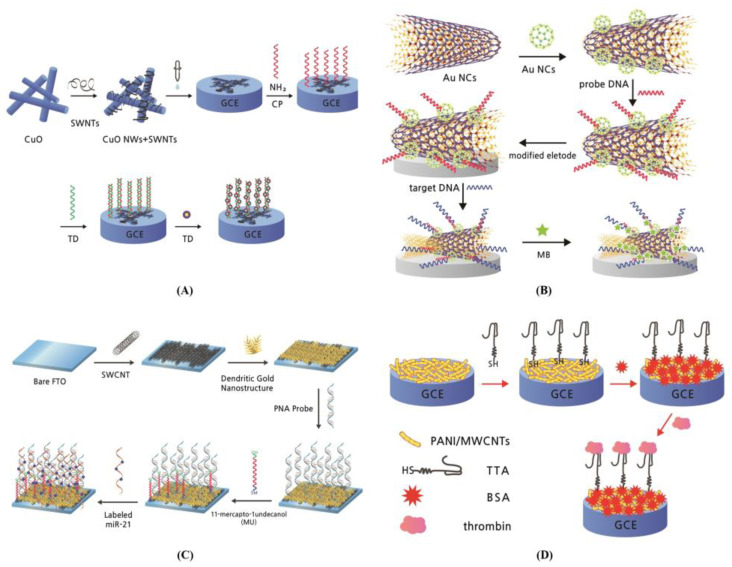
CNT-based biosensor with deoxyribonucleic acid (DNA) enzyme as a bioreceptor including aptamer. (**A**) Schematic process for manufacturing an electrochemical biosensor based on a hybrid nanocomposite consisting of copper oxide nanowires (CuO NWs) and carboxyl-functionalized SWCNTs (SWCNTs-COOH) to detect specific-sequence target DNA [89]; (**B**) Schematic manufacturing process of SPCE electrochemical DNA biosensor [91]; (**C**) Schematic manufacturing process of electrochemical nanogenosensor for highly sensitive detection of miR-21 biomarker [92]; (**D**) Schematic manufacturing process of a biosensor using a nanocomposite consisting of polyaniline and MWCNTs modified with a thiolated thrombin-specific aptamer on a glassy carbon electrode (GCE) for thrombin detection [95].

DNA biosensors are more stable than the antibody-based biosensors and have recently been widely studied. DNA biosensors are applied in many fields, such as environmental monitoring, food control, drug discovery, forensic study, and biomedical research, owing to their excellent sensor performance [98]. The schematics of recent DNA biosensors are described in Figure 7.

Chen et al. developed a sensitive electrochemical biosensor to detect a specific target DNA sequence [89]. The biosensor was composed of a hybrid nanocomposite with copper oxide nanowires (CuO NWs) and carboxyl-functionalized SWCNTs (SWCNTs–COOH). After the manufacturing conditions were optimized, the linear peak currents of Adriamycin were successfully determined with the logarithm of target DNA concentrations ranging from 10^−14^–10^−8^ M and a LOD of 3.5 × 10^−15^ M (S/N = 3) (Figure 7A). Chen et al. further designed an amidated MWCNTs (Au NCs/MWCNTs-NH_2_)-decorated screen-printed carbon electrode (SPCE)-based electrochemical biosensor with high effectiveness and ultra-sensitivity [91]. The SPCE Au NCs/MWCNTs-NH_2_ long non-coding ribonucleic acid (lncRNA) biosensor had a wide linear range of 10^–7^–10^–14^ M and low LOD of 42.8 fM with good selectivity and stability. This MWCNT-based biosensor can detect ln cRNAs for efficient clinical prognosis and cancer diagnosis (Figure 7B). Sabahi et al. manufactured an electrochemical SWCNT-based biosensor for detecting MicroRNA-21 (miR-21) in human blood [92]. This SWCNT-based biosensor was composed of a thiolate receptor-probe functionalized with dendritic gold nanostructures (den–Au) via a self-assembly monolayer (SAM) process and grafted onto the SWCNTs platform on the fluorine-doped tin oxide (FTO) electrode. The oxidation of Cd^2+^ was detected as the sensor signal by differential pulse voltammetry (DPV) containing a broad linear relationship with a miR-21 target molecule concentration of 0.01 fmol/L–1.0 μmol/L and a low experimental LOD of 0.01 fmol/L (Figure 7C). Su et al. demonstrated that a nanocomposite biosensor consisting of polyaniline and MWCNTs modified with a thiolated thrombin-specific Apt (TTA) on the GCE detected thrombin molecule with very low LOD of 80 fM [95]. This MWCNT-based biosensor was manufactured by a thiolene reaction between TTA and chemically synthesized oxidized polyaniline (PANI) in the presence of an MWCNT-dispersed solution. It successfully determined the thrombin in spiked human serum with a molecular level of 0.2–4 nM (Figure 7D).

## 3. CNT-Based Electrochemical Biosensor for Food Safety Assessments

In the era of the fourth industrial revolution, many biosensors for food safety have been developed and commercialized. In particular, the electrochemical biosensors, one of the quick, sensitive, and accurate detectors that monitor the food industry’s safety, including processed foods, can detect food pollutants, allergens, or pathogens that can cause significant health risks when consumed. This biosensor enables quantitative and qualitative analyses, and the advantage of effective real-time monitoring has empowered the practical development of a food analysis technique. However, the current electrochemical biosensor technology has some obstacles, such as delayed analysis time, expensive and complex sample preparation, and instability of sensor signals, which hinder the practical use. Many studies are being conducted to compensate for these obstacles, especially developing an electronic transfer mediator using CNTs, effectively improving sensor signal, analysis time, and cost. CNT-based electron transfer mediators are designed to facilitate electron transfer using various electronic materials, such as metals and organic compounds, and acting as physical or chemical intermediates connecting biosensor receptors. So, these receptors are developed to detect food-related target biomaterials (pathogen, allergen, and other biometabolites), as shown in Table 2.

### 3.1. CNT-Based Biosensor for Food Pathogen Detection

**Figure 8:**
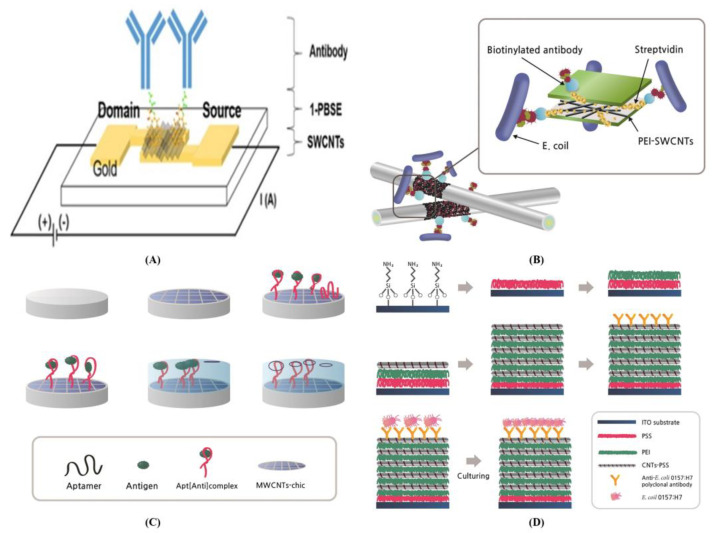
CNT-based biosensors for detecting food pathogen microbes. (**A**) FET biosensor with antibody immobilized with SWCNTs coated with 1-PBASE for the detection of *Staphylococcus aureus* [99]; (**B**) SWCNT-based biosensor with a disposable bio-nano combinatorial junction with gold tungsten wires covered with polyethylenimine (PEI) and SWCNTs for the detection of *Escherichia coli* K-12 [100]; (**C**) Nano-biosensor using Apt [Anti]complex/MWCNTs-Chit/GCE for HCV core antigen detection [102]; (**D**) SWCNTs multilayer biosensor using coupling microfluidic chip-based LAMP for the detection of *E. coli* O157:H7 [103].

Choi et al. developed a FET-based biosensor with antibody immobilized with SWCNTs absorbed with 1- PBASE to detect *Staphylococcus aureus* [99]. The resistance difference (Δ*R*) was calculated using a potentiostat (Figure 8A). The binding of antibodies (pAbs) with S. aureus significantly increased the resistance value of the biosensor (*p* < 0.05), while scanning electron microscope (SEM) images confirmed the specific binding of S. aureus on the biosensor. The SWCNT-based biosensor detected S. aureus with a LOD of 10^4^ CFU/mL in DR of 10^4^–10^7^ CFU/mL.

Yamada et al. [100] integrated SWCNTs and immobilized antibodies into a disposable bio-nano combinatorial junction sensor fabricated to detect *Escherichia* K-12 (Figure 8B). A crossbar junction was formed with gold tungsten wires covered with polyethylenimine (PEI) and SWCNTs, functionalized with streptavidin and biotinylated antibodies for enhancing specificity towards the targeted pathogen bacteria. This biosensor detects the differences in electrical current after the immune reactions. The averaged electrical current increased from 33.13 to 290.9 nA with the presence of SWCNTs in a 10^8^ CFU/mL concentration of *E. coli* K-12. It detected the concentrations of bacterial suspension in the range of 10^2^–10^5^ CFU/mL with a LOD of 10^2^ CFU/mL and a detection time of < 5 min. Ghanbari and Roushani [102] manufactured a nano-biosensor using Apt [Anti]complex/MWCNT-chitosan nanocomposite (Chit)/GCE for hepatitis C virus (HCV) core antigen detection (Figure 8C). Hybrid MWCNT-Chit was used as an immobilized electron transfer mediator (ETM) to improve the conductivity and sensor properties to enhance the loading sensor signal of the Apt sequence. For the first time, the Apt-biosensor was manufactured for the detection of the HCV core antigen. It is beneficial for Apt-sensing and molecular imprinting (MIP) based on the polymerization of dopamine (DA) using electricity for the Apt with HCV core-antigen complex on the MWCNT-Chit-modified GCE. The sensor detected the HCV core antigen via various electrochemical techniques, such as the CV cycle, DPV, and electrochemical impedance spectroscopy (EIS). The Apt sensor can detect a response in the linear range from 5.0 fg/mL to 1.0 pg/mL with a LOD of 1.67 fg/mL. In addition, this study of Apt sensors confirmed the proposed method by determining HCV core antigens in actual human serum samples. SWCNT multilayer biosensor coupling with microfluidic chip-based loop-mediated isothermal amplification (LAMP) was developed by Li et al. (Figure 8D [103]), which can perform visual and point-of-care detection of toxic *E. coli* O157:H7. The anti-*E. coli* O157:H7 functionalized SWCNT multilayer biosensor can select the target pathogen bacteria of *E. coli* O157:H7 among diverse microbes in a sample. The DNA concentration of the released bacteria was subsequently analyzed with microfluidic chip-based LAMP after cleavage of the anti-*E. coli* O157:H7 antibody–bacteria interaction. The LAMP biosensor obtained a significant LOD of 1.0 CFU/mL for *E. coli* O157:H7 with DR of 1.0–10^4^ CFU/mL. Similarly, it had high specificity, low cost, good reproducibility, and the ability to regenerate in related food safety and clinical diagnosis.

### 3.2. CNT-Based Biosensor for Food Allergen Detection

**Figure 9:**
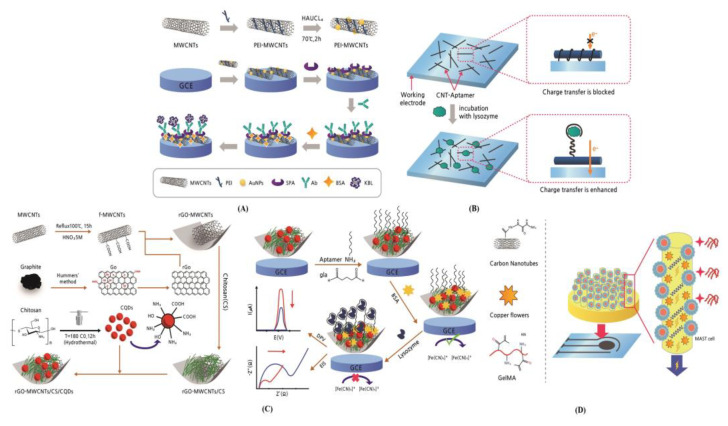
CNT-based biosensors for detecting food allergen proteins. (**A**) An immunosensor modified with AuNPs/MWCNTs/PEI nanocomposite for the allergen assessment of KBL [105]; (**B**) An aptamer-based biosensor using printable ink including a CNT–aptamer complex for the detection of lysozyme [106]; (**C**) An aptamer-based biosensor using GCE/rGO-MWCNT/CS/CQD/APT for the detection of lysozyme [107]; (**D**) A cellular electrochemical biosensor based on FCONp/MWCNT-CDH/GelMA using RBL cell, intestinal microvillus for detecting wheat gliadin, food allergen [108].

Lectins are well-known as allergenic indicators in kidney beans that seriously affect human health. Sun et al. [105] developed a label-free voltametric immunosensor using gold NPs (AuNPs)-polyethyleneimine (PEI)-MWCNT nanocomposite for detecting allergen kidney bean lectin (KBL) with potential activity (Figure 9A).

The KBL polyclonal antibody, acquired from rabbit immunization, was immobilized on the electrode modified with recombinant staphylococcal protein A via the fragment crystallizable (Fc) region of the antibody. Under the optimized condition, this immunosensor obtained an excellent linear response to KBL with a range of 0.05–100 μg/mL, a LOD of 2.30 × 10^−2^ μg/mL, good selectivity, interference-resistant ability, stability (4 °C), and reproducibility. This study provided a rapid quantification of lectin in kidney bean-derived foods and real-time monitoring of the allergenic potential during production and consumption. Khan et al. [106] manufactured an Apt-based biosensor using printable ink, including a CNT–Apt complex for detecting lysozymes as an essential biomarker in various disease diagnoses (Figure 9B). A low-cost immobilization inkjet printing technique is expected to reduce the Apt-based biosensor development cost. The strong winding affinity of the single-stranded DNA onto SWCNTs led to immobilizing the Apts by printing the ink. This Apt-based biosensor obtained a good sensor capacity with a LOD of 90 ng/mL, DR of 0–1.0 µg/mL, and high target selectivity against other proteins. Rezaei et al. [107] developed an Apt-based biosensor using GCE/graphene oxide (rGO)-MWCNT/chitosan (CS)/a synthesized carbon quantum dot (CQD)/Apt for the detection of lysozymes (Figure 9C). This biosensor had a high surface-to-volume ratio, conductivity, stability, and electrocatalytic activity. The amino-linked lysozyme Apts were immobilized on the nanocomposite through covalent coupling between the amine groups of Apt and nanocomposite using glutaraldehyde (GLA). The developed nanocomposite (GCE/rGO-MWCNT/CS/CQD/Apt)-based biosensor accomplished good LODs (3.7 and 1.9 fmol/L) within the wide DRs (20 fmol/L to 10 nmol/L, and 10 fmol/L to 100 nmol/L) for DPV and EIS, respectively. These sensor properties (high sensitivity, reproducibility, specificity, and rapid response) were obtained for lysozyme used in biomedical fields. A cellular electrochemical biosensor based on self-assembled flower-like copper oxide NPs (FCONp)/ MWCNT-cellobiose dehydrogenase (CDH)/gelatin methacryloyl (GelMA) using rat basophilic leukemia (RBL) cells for the intestinal microvillus was manufactured by Jiang et al. [108] for detecting wheat gliadin, a food allergen (Figure 9D). The microvillus structure of the small intestine with cluster formation was printed on the screen-printed electrode (SPE) using stereolithography 3D-bioprinting technology. Similarly, the RBL cells (1 × 10^6^ cells/mL) were immobilized on the gel skeleton for 10 min in the construct of the cellular biosensor. The linear DR is 0.1–0.8 ng/mL, and the LOD is 3.6 × 10^−2^ ng/mL, with good stability and reproducibility, which could be established with potential application in food safety detection and evaluation.

### 3.3. CNT-Based Biosensor for other Food Assessment

**Figure 10:**
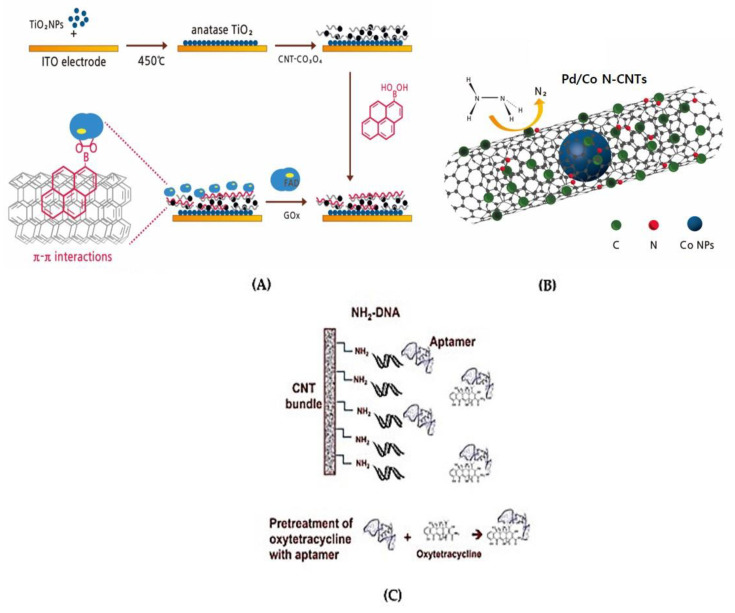
CNT-based biosensor for various biomaterials food assessment. (**A**) A glucose biosensor based on supercapacitor Co_3_O_4_-CNT hybrid on TiO_2_ [113]; (**B**) A hydrazine sensor based on Pd/Co N-CNTs [114]; (**C**) Apt/SWCNT-based electrochemical biosensor for detecting OTC [69].

Cakiroglu and Ozacar manufactured a photoelectrochemical biosensor by blending supercapacitor CNTs with cobalt oxide (Co_3_O_4_) onto the anatase titanium oxide (TiO_2_) deposited on the indium tin oxide (ITO) electrodes (Figure 10A). 

Zhang et al. [114] developed a CNT-based electrochemical biosensor using Pd NPs blended with Co NPs inside of nitrogen-doped carbon nanotubes (Pd/Co-NCNTs) on GCE for the hydrazine detection by CV and EIS (Figure 10B). The Co-NCNTs combined with Pd NPs using pyrolysis led to a high catalytic response and a low overpotential for hydrazine oxidation. The sensor capacity of the Pd/Co-NCNTs/GCE biosensor was a linear DR of 0.05–406.45 μM with an LOD of 7.0 × 10^−3^ μM, demonstrating long-term stability and good sensitivity with a fast response time of at least 2 s. As mentioned before, a simple and highly sensitive Apt-based SWCNT biosensor containing probe-DNA immobilized on functionalized SWCNTs was designed by Yildirim-Tirgi et al. [69] for the detection of OTC and widely employed to resist bacterial infections in livestock and affect their growth rate (Figure 10C). The sensor was fabricated by applying a high-rate nanoscale offset printing process using a directed assembly of SWCNTs with non-covalent functionalization of probe DNA-SWCNTs on two electrodes for the continuous sensor response for OTC antibiotics detection. The developed Apt biosensor had a DR of 10–75 mg/L (20–325 nM) with a LOD of 1.125 mg/L (2.5 nM). Thus, the biosensor was straightforward and fast (less than 10 min for pre-incubation, measurement, and regeneration) with an inherently small size for in vivo applications.

## 4. Conclusions

Biosensor technology can be used in the food industry for tasks such as component analysis, rapid detection of natural toxins and antinutrients, detection of enzyme inactivation and microbial contamination during food processing and storage, measurement of harmful substances produced by the interaction of food ingredients, and determination of the freshness of fish and antioxidant activity. The development of CNTs for detecting biomolecules, one of the most interesting and exciting materials in modern materials’ science, is essential for developing biotechnology in various fields. This review mainly presents the rapidly growing R&D trends of CNT-based electrochemical biosensors. The detection of target molecules is a way to improve various problems such as heat resistance, and the long-term stability, and cost-effectiveness of using nano complexes instead of the fragile enzymes used in the past. When nanocomplex, CNTs, and metal (Au, Pt, etc.) are combined and used, the biosensors’ sensitivity and LOD can be effectively improved.

The advantages of the CNT biosensor are that there is no labeled sign, the device can be miniaturized, the detection time is fast, and real-time detection is possible. However, there are definitely limitations of the CNT biosensor. A representative example is that the hybrid characteristics of metallic/semiconductor properties of CNTs can sometimes interfere with the stability of detection results. However, although the separation technology of metallic and semiconductor properties or selective removal technology of metallic nanotubes has developed a lot to support sensor stability, the increase in unit price or quality in the CNT process should be steadily improved in the future. Another disadvantage is that CNTs are too sensitive, due to poor noise contrast signals. Therefore, the commercialization was no rapidly progressed in recent days thought the related study is progressing in various ways in various fields to develop CNT’s surface treatment technology or signal processing technology. If new nanomaterials and CNTs are combined and continuously developed, these novel materials will be in the spotlight at the molecular level and become a critical scientific challenge. For new target materials in given biomolecules, molecular modeling parallel with experimental studies will quickly pave the way to value-added nano-biosensors. In addition to material engineers, new efforts and active cooperation by chemical, physical, and electrical/mechanical engineers are required in future industries.

## Figures and Tables

**Table 2:** Summary of CNT-based electrochemical biosensors for food assessments.

Target Types	Modified Electrode	Electrical Method		Analyte	LOD	DR	Ref.
Pathogens	SWCNTs/1-PBSE–Ab- *Staphylococcus aureus*	AMP		*S. aureus* in Kimchi samples	10^4^ CFU/mL	10^4^–10^7^ CFU/mL	[99]
	Gold tungsten wires/PEI/SWCNTs –Ab-*E. coli*	AMP		*E. coli* O157:H7	10^2^ CFU/mL	10^2^–10^5^ CFU/mL	[100]
	Amino-modified Apt (ssDNA)/COOH-rich MWCNTs/ITO	CV/IMP		*S. Typhiurium* *S. Enteritidis*	6.7 × 10 *Typhiurium* 5.5 × 10 *Enteritidis* ^(^CFU/mL)	6.7 × 10–6.7 × 10^5^, *Typhiurium* 5.5 × 10–5.5 × 10^6^ *Enteritidis* (CFU/mL)	[101]
	Apt/MWCNTs -Chit/GCE	EIS		HCV in human serum	1.67 fg/mL	5.0 fg/mL–1.0 pg/mL	[102]
	ITO/MWCNTs-PSS/PEI -Ab- *E. coli* O157:H7	EIS		*E. coli* O157:H7	1.0 CFU/mL	1.0 –10^4^ CFU/ml	[103]
Allergen	Au film/CS –MWCNT/GCE	DPV		Ara h1	1.3×10^−17^ mol/L	(3.91–125) × 10^−17^ mol/L	[104]
	Ab-SPA/ AuNPs/PEI_MWCNTs nanocomposite	DPV		KBL in kidney bean milk samples	0.023 µg/mL	0.05–100 µg/mL	[105]
	Printable ink including a CNT–aptamer complex and [Fe(CN)_6_]^−4/−3^ as the redox probe	EIS		Lys	90 ng/mL	0–1.0 µg/mL	[106]
	rGO/MWCNTs/CQDs /CS nanocomposite	DPV/EIS		Lys	3.7 fmol/L (DPV) 1.9 fmol/L (EIS)	20 fmol/L–10 nmol/L (DPV) 10 fmol/L–100 nmol/L (EIS)	[107]
	FCONPs/MWCNTs -CDH/ GelMA	EIS		Gliadin	0.036 ng/mL	0.1–0.8 ng/mL	[108]
	SWCNTs/1-PBSE –Ab-Ara h1 nanocomposite	AMP		Ara h1	1.0 ng/mL	1.0–1000 ng/mL	[109]
	SWCNTs/1-PBSE –Ab-Ara h6 nanocomposite	AMP		Ara h6	10 pg/ml	1.0–10^7^ pg/L	[110]
Other molecules	Cu NPs/Rutin/MWCNTs/ IL/ChI/GCE	CV		H_2_O_2_	0.11 µm	0.35–2500 µM	[111]
	LAC-CNTs-SPCE	EC		Paracresol	0.05 ppm	0.2–25 ppm	[112]
	Co_3_O_4_-SWCNTs/TiO_2_	PLC		Glucose	0.16 µM	0–4 mM	[113]
	Pd/Co-NCNT		CV/EIS	Hydrazine	0.007 µm	0.05–406.045 µm	[114]
	SWCNTs-TFT		TFT	DNA	0.88 µg/L	1.6 × 10^−4^–5 µmol/L	[115]
	SWCNTs/1-PBSE/Apt		EC	OTC	1.125 mg/L	10–75 mg/L	[69]

Abbreviations: Ab: antibody; AMP: amperometry; Apt: Aptamer; Ara h: Arachis hypogea; AuNPs: gold nanoparticles; CDH: cellobiose dehydrogenase; CFU: colony-forming unit; Chit: chitosan; CNT: carbon nanotube; CQDs: carbon quantum dots; CS: chitosan; CV: cyclic voltammetry; DPV: differential pulse voltammetry; DR: detection range; EC: electrochemical; EIS: electrochemical impedance spectroscopy; FCONPs: flower-like copper nanoparticles; FET: field effect transistor; GCE: glassy carbon electrode; GelMA: gelatin methacryloyl; IL: ionic liquid; ITO: indium tin oxide; KBL: kidney beans’ lectin; LAC: laccase; LOD: limit of detection; Lys: lysozyme; MWCNTs: multi-walled carbon nanotubes; MWCNT-CDH: hydrazide-functionalized multiwalled carbon nanotubes; NCNT: nitrogen-doped carbon nanotubes; NPs: nanoparticles; NR: not reported; OTC: oxytetracycline; 1-PBSE: 1-pyrenebutanoic acid succinimidyl ester; PEI: polyethyleneimine; PFU: plaque-forming unit; PLC: photoelectrochemical; PSS: sodium polystyrene sulfonate; RBL: rat basophilic leukemia; Ref.: reference; rGO: reduced graphene oxide; SPA: staphylococcal protein A; SPCE: screen-printed carbon electrode; ssDNA: single strand DNA; SWCNTs: single-walled carbon nanotubes; TFT: thin-film transistor.

## Data Availability

Not applicable.
